# Investigation of Biophysical Mechanisms in Gold Nanoparticle Mediated Laser Manipulation of Cells Using a Multimodal Holographic and Fluorescence Imaging Setup

**DOI:** 10.1371/journal.pone.0124052

**Published:** 2015-04-24

**Authors:** Stefan Kalies, Georgios C. Antonopoulos, Mirko S. Rakoski, Dag Heinemann, Markus Schomaker, Tammo Ripken, Heiko Meyer

**Affiliations:** 1 Biomedical Optics Department, Laser Zentrum Hannover e.V., Hanover, Germany; 2 Department of Cardiothoracic, Transplantation and Vascular Surgery, Hannover Medical School, Hanover, Germany; University of Zurich, SWITZERLAND

## Abstract

Laser based cell manipulation has proven to be a versatile tool in biomedical applications. In this context, combining weakly focused laser pulses and nanostructures, e.g. gold nanoparticles, promises to be useful for high throughput cell manipulation, such as transfection and photothermal therapy. Interactions between laser pulses and gold nanoparticles are well understood. However, it is still necessary to study cell behavior in gold nanoparticle mediated laser manipulation. While parameters like cell viability or perforation efficiency are commonly addressed, the influence of the manipulation process on other essential cell parameters is not sufficiently investigated yet. Thus, we set out to study four relevant cell properties: cell volume and area, ion exchange and cytoskeleton structure after gold nanoparticle based laser manipulation. For this, we designed a multimodal imaging and manipulation setup. 200 nm gold nanoparticles were attached unspecifically to canine cells and irradiated by weakly focused 850 ps laser pulses. Volume and area change in the first minute post laser manipulation was monitored using digital holography. Calcium imaging and cells expressing a marker for filamentous actin (F-actin) served to analyze the ion exchange and the cytoskeleton, respectively. High radiant exposures led to cells exhibiting a tendency to shrink in volume and area, possibly due to outflow of cytoplasm. An intracellular raise in calcium was observed and accompanied by an intercellular calcium wave. This multimodal approach enabled for the first time a comprehensive analysis of the cell behavior in gold nanoparticle mediated cell manipulation. Additionally, this work can pave the way for a better understanding and the evaluation of new applications in the context of cell transfection or photothermal therapy.

## Introduction

The emerging field of nanomedicine aims to diagnose and treat various diseases with nanostructures from a hundred down to a few nanometers [1]. Many applications in nanomedicine utilize gold nanoparticles. In combination with biomolecules and surface treatment, nanoparticles are used to deliver various agents, e.g. nucleotides, to cells [2, 3]. Nanoparticles provide special properties compared to bulk material, such as different optical absorption properties [2]. Today, the interaction of gold nanoparticles and light is frequently used for labeling cells or as biosensors for microscopy.

Many recent studies focus on the use of lasers as light sources and gold nanoparticles to manipulate cells [4–7]. Huang et al. demonstrated applications of this configuration for plasmonic photothermal therapy [4, 5]. In this approach, gold nanoparticles served as photothermal contrast agents and their heating was used to eliminate cell carcinoma. Lukianova-Hleb employed so called plasmonic nanobubbles generated around gold nanoparticle clusters trough irradiation with short laser pulses to eliminate cells in a monolayer [7, 8]. Our group uses gold nanoparticles irradiated by picosecond laser pulses for high-throughput delivery of antisense molecules or proteins in a technique called gold nanoparticle mediated laser transfection [6, 9].

Intense research has been conducted concerning the interactions of nanoparticles and the electric field of the irradiating laser. Depending on the properties of the gold nanoparticles and the laser parameters, possible interactions include heating and near field enhancement [10]. Both processes can lead to bubble formation [11–13].

The interaction between cells and irradiated gold nanoparticles as a complete system on single cell level is yet not well understood. Most studies investigate applications of gold nanoparticle mediated laser manipulation by studying uptake of dyes or biomolecules in cell monolayers [6, 13]. This study addresses the important question of how a cell reacts to laser irradiated gold nanoparticles. We focus on laser and particle parameters similar to those used in gold nanoparticle mediated laser transfection, because possible particle interactions, cell viability, and dye uptake were already analyzed in our previous publications [6, 9, 14, 15]. Moreover the investigation of higher laser powers enables the analysis of cell killing as in photothermal therapy. We examined four essential cell parameters after gold nanoparticle mediated laser manipulation: cell volume and area, filamentous actin (F-actin) cytoskeleton structure, and ion concentration.

In order to characterize the exchange of cytoplasm and extracellular medium after gold nanoparticle mediated laser manipulation, the examination of cell morphology is of importance. Digital holography enables us to calculate the change in cell volume as well as cell area after laser manipulation [16–18]. Antkowiak et al. followed a similar approach in the case of femtosecond laser transfection with direct focusing on the cell membrane [19]. The cytoskeleton is related to a cell’s mechanical structure and stability. One major component of the cytoskeleton is microfilaments composed of actin. Analyzing the F-actin distribution can produce new insights regarding cell damage and stress due to gold nanoparticle mediated laser manipulation. Calcium is an essential parameter which is commonly used to evaluate changes in the ion concentration of a cell. Membrane perforation may lead to ion exchange with extracellular medium. Additionally, a variety of intracellular pathways, such as IP_3_, can lead to ion increase or decrease [20]. Furthermore IP_3_ and paracrine signaling can evoke intercellular calcium waves [20–23]. This has previously been observed in femtosecond irradiation of cells [24].

In order to further analyze the processes involved in gold nanoparticle mediated laser manipulation, we applied and visualized fluorescent markers for calcium level and F-actin distribution. We built a setup which combines fluorescent and holographic imaging during irradiation of the gold nanoparticle labeled cells with picosecond laser pulses. This furthermore enables us to analyze the suitability of digital holography in conjunction with gold nanoparticle mediated laser manipulation. The combination of fluorescence and digital holographic imaging enabled us to correlatively analyze morphological and physiological parameters.

## Materials and Methods

### Multimodal imaging and manipulation setup

**Fig 1:**
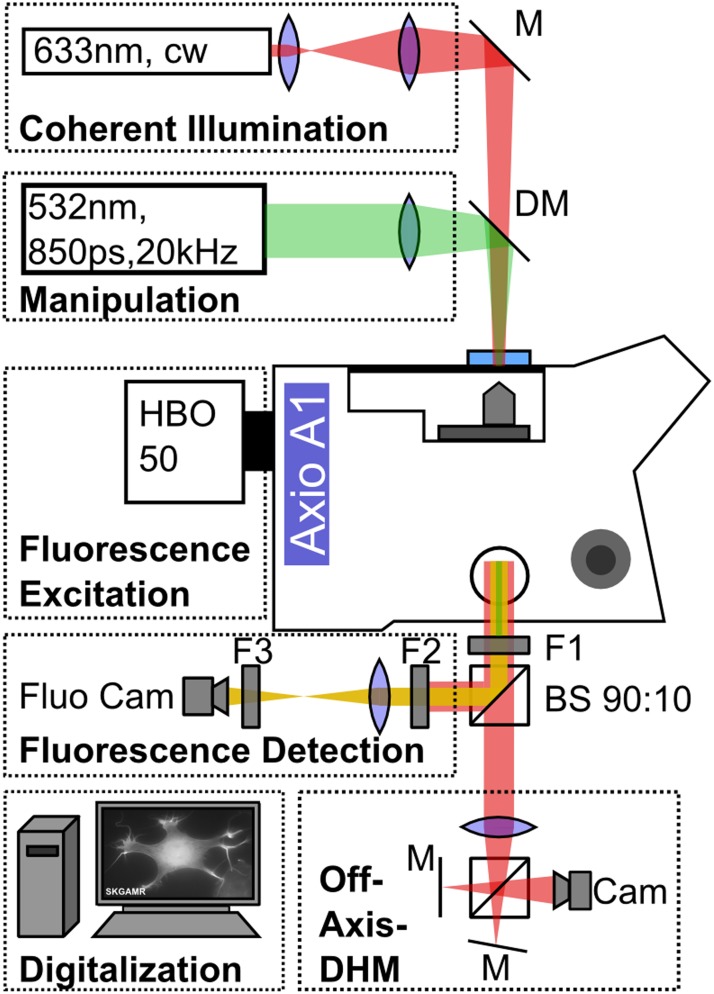
Schematic of the experimental setup. An epifluorescence microscope is modified to capture digital holography and fluorescence image data. Coherent illumination necessary for digital holography is realized by weakly focusing a cw Helium Neon Laser on the sample. The pulsed manipulation laser is coupled into the setup parallel to the HeNe-Laser using a dichroic mirror (DM) and focused onto the sample. For fluorescence excitation a mercury vapor lamp is employed. At the side port of the microscope the manipulation laser is attenuated using a notch filter (F1). A beam splitter directs 90% of the light to the fluorescence unit where it passes through a filter blocking the coherent illumination (F2) and an emission filter (F3). The remaining light travels to the digital holography module which is set up in off-axis Michelson configuration.

A commercial epifluorescence microscope (Axio Observer.A1, Carl Zeiss, Germany) was modified to add quantitative phase imaging modality using off-axis digital holography, as shown in Fig 1. Cell dishes were placed in an incubation chamber (okolab, Italy) that was mounted on the motorized xy-translation stage (ProScan II, Prior Scientific, UK) of the microscope. During the imaging and manipulation process the lids of the cell dish and the incubation chamber were removed, but heating to 37°C and gas flow at 5% CO_2_ were left operative. Imaging was performed with a 63x microscope objective with NA = 1.40 (Plan-Apochromat 63x/1.40 Oil M27, Carl Zeiss, Germany).

Off-axis digital holography requires illumination of the sample with coherent light. We loosely focused a cw-Helium Neon Laser (05-LHP-111, *λ* = 632.8 nm, P = 1.3 mW, Melles Griot, USA) onto the sample from above and adjusted the spot size to illuminate the field of view of the microscope objective. For brightfield microscopy an incoherent illumination was realized with an amber LED (Luxeon Rebel Amber LXML-PL01-0050, Philips Lumileds, USA). For cell manipulation, a 532 nm pulsed Nd:YAG microchip laser (Horus, France) with a pulse duration of 850 ps and repetition rate of 20.25 kHz was coupled into the illumination beam path using a dichroic mirror. The laser was focused on gold nanoparticle labeled cells with a spot diameter of approximately 80 *μ*m. Irradiation time for the manipulation laser was controlled using a mechanical shutter (SH05, Thorlabs, USA). Laser power was adjusted using an attenuation unit. Fluorescence illumination was performed with a mercury vapor lamp (HBO 50, Carl Zeiss, Germany).

At the side port of the microscope the manipulation laser beam was attenuated with an OD6 notch filter (NF533-17, Thorlabs). Using a non-polarizing beamsplitter (BS028, Thorlabs), 90% of the remaining light was reflected into the fluorescence detection unit and the remaining 10% were transmitted to the digital holography setup. For fluorescence imaging, light from the sample first passes a notch filter (NF633-25) for suppression of the HeNe laser and then passes a fluorescence filter to detect the desired fluorophore emission. Images were captured by a cooled CCD fluorescence camera (ProgRes MF cool, JENOPTIK, Germany). The digital holography unit was a simplified off-axis setup as described by Kemper et al. consisting of a beamsplitter and two tilted mirrors [17]. The image formed at the side port was captured with a CMOS camera (SME-B050-U, Mightex Systems, Canada).

### Cell culture and preparation

ZMTH3 cells, kindly provided by Murua Escobar et al., were cultured in RPMI 1640 supplemented with 10% fetal calf serum and the antibiotics penicillin and streptomycin (all Merck, Germany) at 37°C in a humidified 5% CO_2_ atmosphere [25]. In order to generate F-actin labeled cells, ZMTH3 cells were transfected with the plasmid pLifeAct-mTurquoise2 (Addgene plasmid 36201, Addgene, US), kindly provided by Dorus Gadella [26] using Roti-Fect (Carl Roth, Germany) transfection reagent. Transfected cells were selected and maintained in medium supplemented with 1000 *μ*g/mL of the antibiotic G418 (Merck) and termed ZMTH3-Act cells. The pLifeAct-mTurquoise2 plasmid encodes a marker for the visualization of F-actin in living cells [27].

All experiments were performed in 35 mm culture dishes (ibidi, Germany). Cells were seeded at a density of 10,000 cells for digital holography measurements and 100,000 cells for all other experiments on the day before the experiment. Spherical gold nanoparticles (PGO-200, Kisker Biotech, Germany) with a size of 200 nm and a surface concentration of 0.5 *μ*g/cm^2^ were added to the ZMTH3 cells three hours before laser manipulation [6]. Directly before laser manipulation, the medium containing the particles was replaced with fresh culture medium without phenol red.

Cell perforation and viability were assessed within thirty laser irradiated areas of three independent dishes (see S1 Fig). Holographic microscopy and calcium imaging experiments were performed in three independent samples each with ten and five single cells, respectively. F-actin was analyzed with ten irradiated cells in total. The laser parameter set consisted of three radiant exposures (15, 27, and 41 mJ/cm^2^) at two irradiation times (10 and 40 ms). Radiant exposure was calculated as the quotient of pulse energy and spot area [6, 12]. Statistical significance was tested by applying Student's t-test against the control group. Significance levels are indicated using stars as follows: * means *p* < 0.05, ** means *p* < 0.01 and *** means *p* < 0.001, while a result is considered not statistically significant for *p* > 0.05 and marked ns. Stars are depicted in figures where a t-test was performed for the dataset.

### Digital Holography

#### Obtaining quantitative phase images

The detailed process of obtaining quantitative phase information (QPI) from interferometric data is presented elsewhere (see, e.g. [28]) and shall be described only briefly. The off-axis digital holography setup above obtains interferograms by superimposing a wavefront with a tilted copy. When imaging a cell, the tilt has to be such, that this cell overlaps with a sample free area [17]. Fringe analysis and subsequent phase unwrapping were implemented in C++ and carried out on high end desktop computers (Intel Core i5-4570 CPU, 32GB DDR3 RAM). Phase unwrapping was performed using the SRNCP algorithm [29]. Residual wavefront aberrations and constant background were subtracted by fitting a second-order polynomial to the background using a semi automated custom ImageJ macro.

The wavefront phase *ϕ*(*x*, *y*) is related to cell morphology via the optical path length *L*_*opt*_ by *ϕ*(*x*, *y*) = 2*π*/*λ*⋅*L*_*opt*_(*x*,*y*) [19]. An optical path length difference *δL*_*opt*_ is observed between sample free areas and the cell. Let *D* be the thickness of the medium layer, and *d*(*x*,*y*) the thickness of the cell at a point (*x*,*y*) and *n*_*med*_ and *n*_*cell*_ be the refractive indices of the medium and the cell respectively, then

$$\begin{array}{ccc} \Delta L_{opt}\left(x,y\right) & = & \left(D-d\left(x,y\right)\right)\cdot n_{med}+d\left(x,y\right)\cdot n_{cell}\left(x,y\right)\\ & = & d\left(x,y\right)\cdot (n_{cell}\left(x,y\right)-n_{med})+const. \end{array}$$

The constant is zero after background is removed in the image. In the equation above it is already presumed that the refractive index *n*_*cell*_ varies only slowly over the height of the cell [30]. While *n*_*med*_ = 1.34 was measured with an Abbe refractometer, the exact refractive index of ZMTH3 cells is unknown. We ultimately analyzed relative changes in cell phase volume and area, so the exact values of the refractive indices were not needed. Calculating the integral of the optical path lengths at every point within the cell area *A* gives the phase volume of the cell:

$$\begin{array}{c} V_{ph}=\int_{A}\text{dxdy}\;\Delta L_{opt}\left(x,y\right) \end{array}$$

Some care has to be taken when interpreting the cell phase volume as a morphological property of the cell, since both the refractive index of the cell and cell thickness factor into it [31, 32]. A change in protein concentration in the cell can affect its refractive index. In this case, it is difficult to separate cell volume from cell refractive index. An inflow of medium alone will effect no measurable change in the phase volume since no additional optical path length difference will be induced. However, if a change in cell volume is dominantly caused by outflow of cytoplasm into the surrounding medium, then the main contribution to the change in *V*_*ph*_ can be attributed to a change in cell thickness. In this case, *V*_*ph*_ is proportional to cell volume.

#### Cell phase volume after irradiation

Single cells were captured with a frame rate of 33 fps with a pixel resolution of 1280×1024 for a total of 66 s. Cells were irradiated approximately 1 s after capture start. Cell phase volume was calculated as the discrete integral of the cell height using a custom ImageJ macro and normalized to the phase volume of the cell pre irradiation. We examined the cell phase volume directly after laser exposure. The normalized phase volume was analyzed by least squares fitting of an exponential-linear model. If exponential-linear fits failed either due to non-convergence or if parameter errors exceeded the parameters, a linear model alone was used. Plots and fits were produced using Origin 9.1G (OriginLab, USA). At least 22 of 30 cells for each parameter set were evaluated. Singular cells had to be excluded due to imaging artifacts or reconstruction failure.

#### Cell area after irradiation

Cell area was measured 30 s and 60 s after irradiation and then normalized to the initial area. Measurements were performed manually by selecting the cell boundary and calculating the included area using ImageJ. This procedure was performed on downsampled versions of the time series (xy scaling factor 0.33, time scaling 1/50).

### Fluorescence Imaging

#### Viability and perforation efficiency

The viability of the cells was evaluated by a life-dead assay using calcein AM (acetoxymethyl) green (1 *μ*M, life technologies, USA) and 1.5 *μ*M of the membrane impermeable dye propidium iodide (life technologies) one hour post laser manipulation. After propidium iodide binds to nucleic acids within the cell, its fluorescence can be detected. Esterases in living cells hydrolyse the acetoxymethyl of calcein AM yielding a green fluorescent calcein signal. Cells stained calcein positive and propidium iodide negative were identified as viable and counted as described above (see S1 Fig).

The perforation efficiency was studied in a separate experiment. Propidium iodide was added before laser manipulation at a concentration of 1.5 *μ*M as also described by Antkowiak et al. and Mitchell et al. [19, 33]. Here, propidium iodide enters the cell due to membrane perforation. Its fluorescence was assessed one minute after perforation. All perforated cells within the area of the laser spot were counted manually using ImageJ [34]. Perforation efficiency was calculated as the product of perforated and viable cells. The filter for calcein excitation was a 480 nm ± 15 nm bandpass filter, emission was evaluated using a 520 nm longpass filter. Propidium iodide was excited using a 535 nm ± 20 nm filter and detected with a 610 nm ± 25 nm bandpass filter. A control group containing cells without gold nanoparticles was irradiated separately and no inflow of propidium iodide was observed. Triton X-100 was applied to untreated cells to cause propidium iodide inflow as a positive control.

#### Calcium response

The calcium signal was assessed by the intensity of the dye Fluo-4 (life technologies). The Fluo-4 AM form was used to load the cells. Cells were washed three times with medium without any supplements. Fluo-4 AM was added to the cells at a concentration of 1.8 *μ*M and cells were incubated for 20 min at 37°C. Subsequently, the medium was replaced with fresh supplement-free medium and cells were again incubated for another 20 min. In a further experiment, free calcium in RPMI was buffered by 5 mM EGTA [35]. The fluorescence of the Fluo-4 signal was excited at 475 nm ± 35 nm, recorded at 545 nm ± 30 nm, and normalized to its initial value by manually selecting the cell boundary using ImageJ. A selection containing no cells was subtracted as background. The frame rate of the recording was 5 fps with 200 ms exposure time using linear camera gain. In each irradiated area a single cell was analyzed.

#### Visualization of the F-actin cytoskeleton

F-actin was analyzed in the ZMTH3-Act cells with the marker-plasmid for F-actin. The chosen excitation wavelength of mTurquoise2 was 475 nm ± 35 nm and emission was evaluated at 545 nm ± 30 nm. Nonlinear gain settings as well as a comparably long exposure time of 500 ms were used. We visualized F-actin for 10 minutes post laser manipulation with one frame per minute. To analyze F-actin reorganization after gold nanoparticle mediated laser manipulation, we followed an approach recently used by Weichsel et al. and Chen et al. [36, 37] and quantified the level of orientation of F-actin fibers in a cell. This was done using the ImageJ plugin OrientationJ [38, 39], that allows to measure the coherency of local image features. A coherence value of 1 indicates a dominant orientation of local image features, while a coherence of 0 indicates no orientation [39]. Usually, a well-organized F-actin pattern would yield a high coherency. For each point in time we measured the total coherency, that is the sum of coherency values, in the area of a cell. For each cell, this value was then normalized to the total coherency pre irradiation to obtain the normalized total coherency.

## Results and Discussion

### Perforation efficiency and cell viability

**Fig 2:**
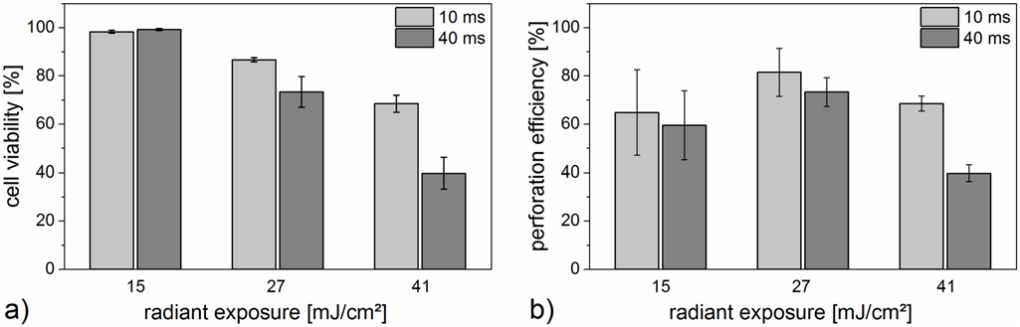
Cell viability (a) and perforation efficiency (b) for different radiant exposures and irradiation times. Viability was evaluated with a calcein/propidium iodide assay one hour after perforation. The perforation efficiency was analyzed via uptake of propidium iodide one minute after laser perforation and is the product of perforated and viable cells. It decreases with higher radiant exposures. Highest perforation efficiency is achieved with 10 ms exposure time and 27 mJ/cm^2^. The mean and standard deviation of three independent measurements are shown. Each measurement consisted of thirty irradiated cell areas.

The irradiation times and radiant exposures we studied resulted in cell viabilities ranging from about 40% up to 95%, see Fig 2 a) and S1 Fig a). In this case propidium iodide served as an indicator of cell death by measuring the membrane integrity one hour after laser manipulation. Cell viability decreased with increasing radiant exposure, showing minimum observed viability at the highest values for irradiation time and radiant exposure.

The perforation efficiency of the cells varied from 40% to 80%. Highest perforation efficiency for both irradiation times was observed for a radiant exposure of 27 mJ/cm^2^. The perforation efficiency is the product of perforated and viable cells. Thus, although nearly all cells showed uptake of propidium iodide after perforation with 41 mJ/cm^2^ and 40 ms (see Fig 2 b), S1 Fig b), and S2 Fig), only viable cells were counted as successfully perforated. Irradiated cells without gold nanoparticles did not show perforation.

In some cases, radiant exposures of 27 mJ/cm^2^ and 41 mJ/cm^2^ led to the formation of membrane blebs, which was visible with the propidium iodide inflow. These blebs might suggest a locally high intracellular pressure and could also be associated to cell death [40]. However, blebbing can potentially be reversible and blebs can be retracted by the actin cortex in some cases [41].

### Holographic measurement of cell phase volume and area

We applied digital holography to assess the time dependency of phase volume and area of cells after gold nanoparticle mediated laser manipulation. Due to limited spatial and temporal resolution, we did not aim to observe membrane poration directly. Instead our objective was to determine the behavior of the whole cell in the first minute post perforation. This timespan is sufficient for molecular delivery as evidenced by propidium iodide inflow.

**Fig 3:**
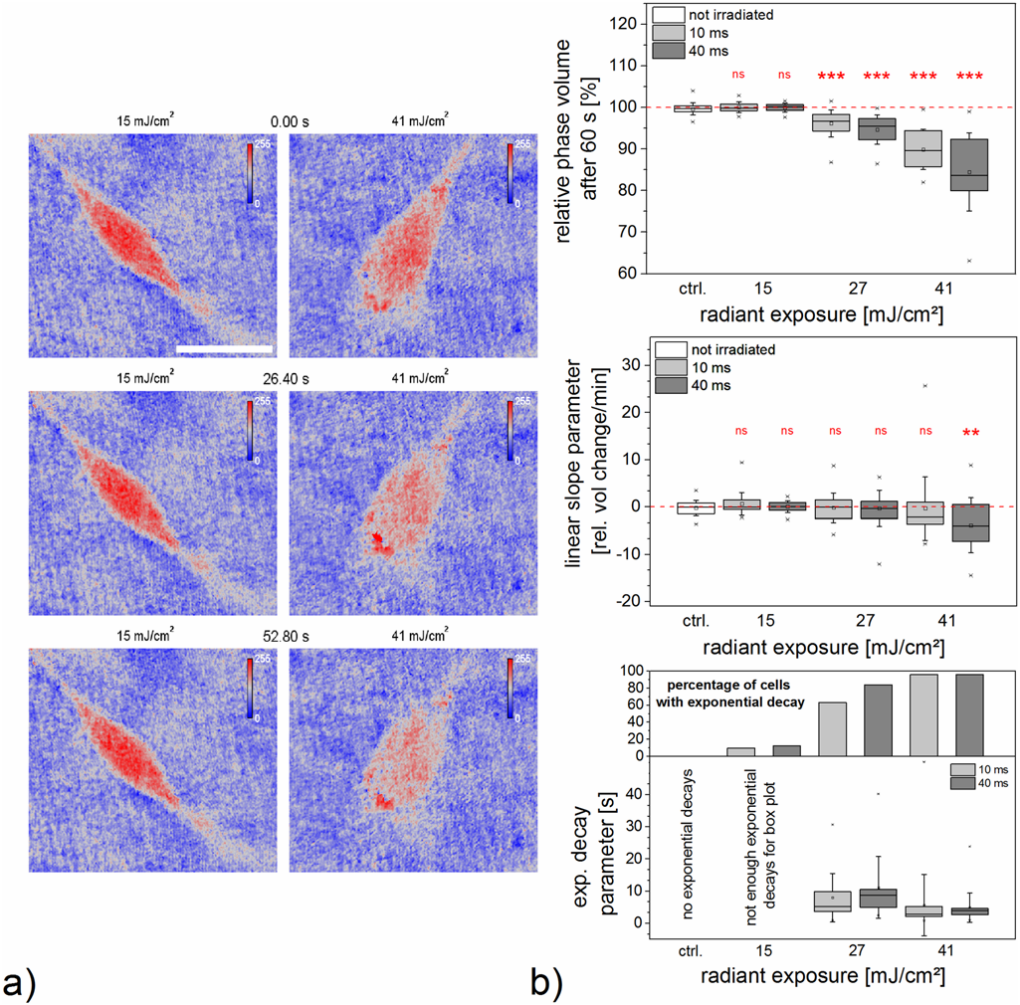
Cell phase volume change evaluated by holographic microscopy. a) Phase images show phase volume and area change after 40 ms irradiation time for two radiant exposures. See corresponding supporting information S1 Video. Scale bars are 20 *μ*m. b) Shown here are box plots of the fit parameters for the relative phase volume change of cells over a time of 60 s. See supporting information S3 Video. Whiskers for box plots depict one standard deviation. The upper box plot indicates that the total loss of phase volume increases with radiant exposure. The middle box plot presents the linear slope of the fit, which is an indicator of the slow process of cell phase volume change. It can be either positive or negative, corresponding to linear phase volume increase or decrease. In the bottom box plot, the exponential decay parameter is depicted. The percentage of cells, which show a combination of linear and exponential decay is indicated in the upper histogram panel. The graph below this shows the decay constants for those cells which exhibited an exponential volume decay after irradiation. The decay parameter is smallest on average for highest radiant exposure, suggesting fastest loss of volume. Data of at least 22 irradiated cells is shown.

In order to evaluate phase volume as a function of time, we normalized the cell phase volume after irradiation to its initial value pre irradiation. In the first 5–10 seconds after irradiation, cells either showed a fast phase volume decrease or it remained nearly constant over the whole 60 seconds (see Fig 3 and see supplementary information S3 Fig and S1 Video). No fast phase volume increase was observed. After the fast phase volume decay most cells showed a further slow gain or loss of phase volume. To characterize the normalized cell phase volume *V*_*ph*_(*t*) as a function of time we used a simple model that was least squares fitted to all data:

$$\begin{array}{c} V_{ph}\left(t\right)=\underset{\text{exp.}\;\text{decay}}{\underset{︸}{a\cdot \exp(-\ln2\cdot t/\tau )}}+\underset{\text{lin.}\;\text{behavior}}{\underset{︸}{m\cdot t}}+\;c \end{array}$$

The fast decay is modeled by the exponential part of the equation. An exponential decay can be used to model cell volume changes due to regulatory effects, osmosis or flow [42, 43]. The exponential decay parameter *τ* is the half life time of the initial fast volume decay. The linear term describes the slow phase volume change that dominates asymptotically towards the end of the time series. Using a linear model is in agreement with the data and is the simplest model for any underlying process within the time span of observation. The linear slope parameter *m* represents the rate of relative phase volume change at the end of the time series and can be positive, negative or zero. A nonzero linear slope parameter indicates a process that affects phase volume in a timespan larger than the measured interval. As a derived result, the phase volume after 60 seconds was calculated as *V*_*ph*_(*t* = 60*s*). Fig 3 gives an overview of all three evaluated parameters.

Almost independent of the irradiation time, the change in cell phase volume was most pronounced with a high decrease for the highest radiant exposure of 41 mJ/cm^2^ (see Fig 3 b)). Compared to this, a less noticeable change in phase volume was observed using 27 mJ/cm^2^. For both radiant exposures, the decrease in volume was significant compared to the unirradiated control group (*p* < 0.001). A radiant exposure of 15 mJ/cm^2^ yielded virtually the same results as an unirradiated control group (*p* ≥ 0.17). In these groups, linear phase volume increase and decrease were zero on average. An exponential decay part was not observed in the control group and existed only in two samples in the group of 15 mJ/cm^2^.

For a radiant exposure of 41 mJ/cm^2^, most obvious and statistically significant in the case of 40 ms (*p* < 0.01). The median of the linear slope parameter indicated a rate of phase volume change of 5% of the initial cell phase volume. The median of cells for 27 mJ/cm^2^ had a linear slope parameter of nearly zero (see Fig 3 c)) which did not significantly differ from the control group (*p* ≥ 0.91). An exponential decay part was observed for radiant exposures of 27 mJ/cm^2^ and 41 mJ/cm^2^ with an occurrence frequency of more than 60% (see Fig 3 d)). For 41 mJ/cm^2^, exponential decay was faster, as indicated by a lower exponential decay parameter *τ*.

**Fig 4:**
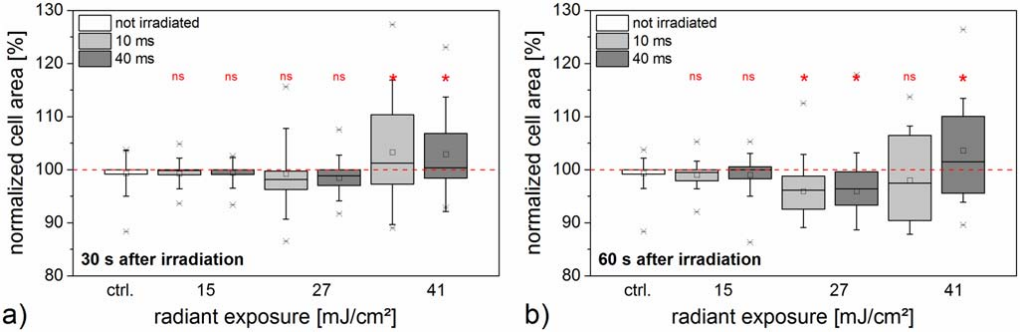
Cell area evaluated by holographic microscopy after laser manipulation. Cell area a) 30 s and b) 60 s post irradiation is normalized to the cell area before laser manipulation. Box plots indicate the distribution of the area for the irradiation times and radiant exposures under examination. The unirradiated control is based on the same dataset in both diagrams. The highest radiant exposure of 41 mJ/cm^2^ led to a diversified area distribution, while 27 mJ/cm^2^ mainly caused an area decrease. In the case of 15 mJ/cm^2^ the area distribution was similar to irradiated cells. Data of at least 22 irradiated cells is shown.

Moreover, we analyzed the change in cell area at two distinct points in time of 30 s and 60 s post laser manipulation (see Fig 4). The control group and the lowest radiant exposure of 15 mJ/cm^2^ showed no significant change in area compared to the control group (*p* ≥ 0.66). In the case of 27 mJ/cm^2^, a decrease in area was observed for most cells, which was significant 60 s after laser irradiation (*p* < 0.05). On average, the shrinkage continued from 30 s to 60 s. A radiant exposure of 41 mJ/cm^2^ yielded a heterogeneous change in area, with a tendency of area increase at 30 s.

As evidenced by the intracellular fluorescence of propidium iodide after manipulation in the previous section, it is safe to assume an inflow of molecules into the cell. In digital holography measurements, we observed mostly phase volume and area decrease, in particular for the higher radiant exposures of 27 mJ/cm^2^ and 41 mJ/cm^2^. We believe that the initial fast volume decay is indeed primarily caused by a cell volume change due to the outflow of cytoplasm, rather than a refractive index change. This is supported by the cell morphology during the time series measurement and the fact that the initial phase volume decay can be modeled with an exponential function. We believe that these morphological changes are related to cellular stress. This raises the question of how in- and outflow are related during and post laser manipulation. The perforation process leads to membrane opening, e.g. pore formation. This can either be reversible or irreversible and produces an exchange of volume. Diffusion through these openings can induce in- and outflow of molecules, therefore propidium iodide positive staining of the cell occurs. It can occur passively by a flow through the membrane opening during cell shrinkage. The outflow can occur within a short timescale (see 41 mJ/cm^2^ in Fig 3) and can exceed the inflow. A high loss of volume within a short time can lead to cell death (see Fig 2). The cell area exhibited decrease as well as increase for the total of cells in this case (see Fig 4). With lower outflow coefficients more cells can survive (see 27 mJ/cm^2^) and the loss in volume is associated with a loss in area. In the case of 15 mJ/cm^2^ the outflow might balance the inflow or the cell stress has no morphological consequences. Propidium iodide outflow is not visible in the fluorescence microscope. Its fluorescence is only visible when it is bound to nucleic acids (RNA and DNA) which are probably too large to flow out of the cell.

### Calcium signal after gold nanoparticle mediated laser manipulation

In addition to holographic measurements of the cell phase volume and area, we studied the ion exchange during gold nanoparticle mediated laser manipulation. In particular, we addressed the calcium response in cells post laser manipulation with the fluorescent calcium indicator Fluo-4.

**Fig 5:**
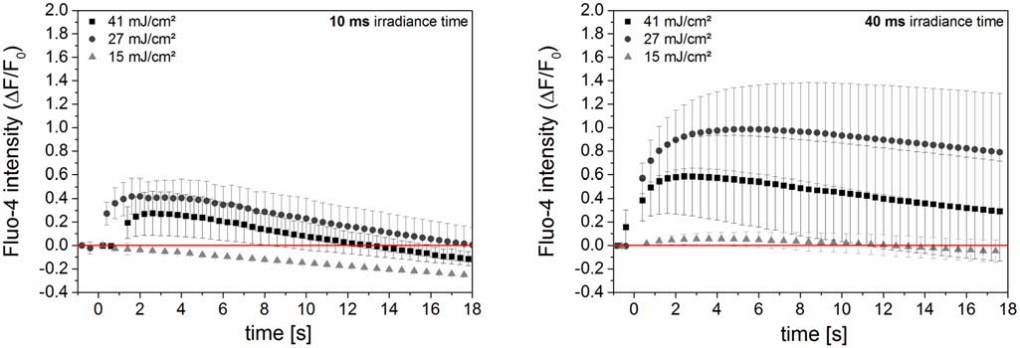
Normalized fluorescence of calcium indicator Fluo-4 after laser perforation. Fluorescence intensity is normalized to the value before laser manipulation. The point in time of laser manipulation was set to zero seconds. The increase of Fluo-4 fluorescence intensity after laser manipulation corresponds to an increase in intracellular calcium, see Fig 7, as well as inflow of extracellular calcium. The process is highly dependent on radiant exposure and irradiation time. Signal decrease below base level is most likely explained by photobleaching. The mean and standard error of three experiments with five irradiated and analyzed cells are shown.

We examined the fluorescence level of Fluo-4 in cells before and over a timespan of 18 s post laser manipulation (see Fig 5). The Fluo-4 signal was evaluated relative to its pre-laser value and is an indicator of calcium level changes within the cell. The maximum increase of the Fluo-4 signal was higher for 40 ms irradiation time for all radiant exposures compared to 10 ms. It was most pronounced for a radiant exposure of 27 mJ/cm^2^. The decrease in peak response for the higher radiant exposure of 41 mJ/cm^2^ might be caused by the cell volume outflow that is observed with digital holographic measurements.

**Fig 6:**
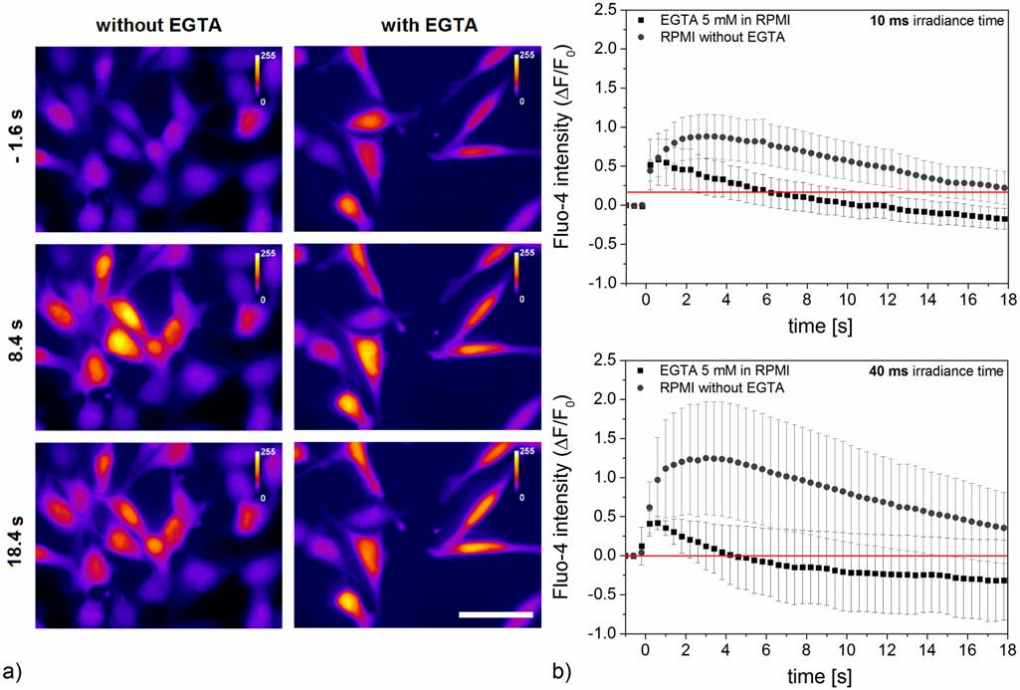
Normalized fluorescence of calcium indicator Fluo-4 with and without addition of calcium chelator EGTA. a) Fluorescence images at different points in time referring to supplemental video S2 Video. Scale bar 50 *μ*m. The color map calibration is different for both columns. b) Time-dependence of Fluo-4 intensity relative to its initial value after laser manipulation at zero seconds. 5 mM EGTA was used as calcium chelator in RPMI 1640. Radiant exposure was fixed to 27 mJ/cm^2^. The peak increase with 5 mM EGTA compared to normal conditions is lowered. Signal decrease below base level is most likely explained by photobleaching. The mean and standard error of three experiments with five irradiated and analyzed cells are shown.

To analyze the influence of extracellular calcium, which might enter the cell during perforation, we applied 5 mM EGTA as a calcium chelator before laser manipulation. We used a fixed radiant exposure of 27 mJ/cm^2^ because this corresponded to the highest measured perforation efficiency. Based on the initial value of Fluo-4 intensity, the peak intensity was weaker in the samples containing 5 mM EGTA compared to RPMI 1640 without additive (see Fig 6 and S2 Video). Moreover, we examined the distribution of peak amplitude and the corresponding time at which it was reached (see S4 Fig). While the peak amplitude was similar in presence or absence of EGTA, a decrease in the duration until peak amplitude in the presence of EGTA occurred. Additionally, an intercellular calcium wave in adjacent but non irradiated cells was still observed.

**Fig 7:**
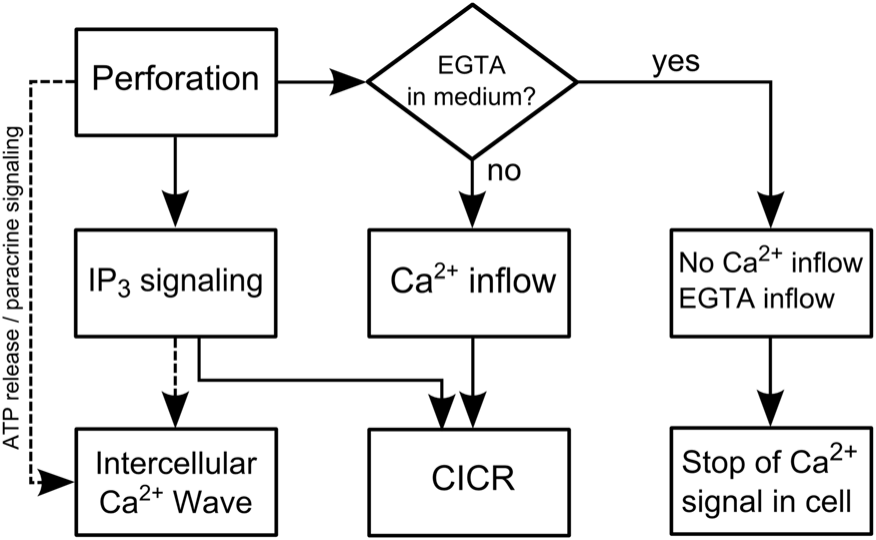
Potential causes of the calcium signaling during gold nanoparticle mediated laser manipulation. Two different pathways in the presence or absence of a calcium chelating agent like EGTA are depicted. The intercellular calcium wave occurs in the presence of EGTA because the cascade is possibly mediated by the IP_3_ pathway or paracrine ATP signaling. ATP might also be released from the cell after perforation.

The extracellular calcium level is usually about four orders higher than inside the cell. Extracellular ions can enter the cell after perforation, which yields an increase in calcium. An intracellular pathway which can lead to calcium increase is the IP_3_ (inositol 1,4,5-triphosphate) signaling pathway [20, 21]. The IP_3_ pathway can be triggered by membrane stress due to laser induced membrane poration or heating [20, 24, 44]. By binding of IP_3_ to an IP_3_ receptor, intracellular calcium release is stimulated. Additionally, the elevated calcium levels can further induce calcium release through CICR (calcium stimulated calcium release) via the IP_3_ pathway in a self-limited process [45]. After the addition of EGTA, extracellular calcium is chelated and no increase due to inflow is possible. The observed signals are therefore most likely partly associated with the IP_3_ pathway. However, EGTA (0.38 kDa) can enter the cell during perforation and intracellular calcium might be chelated after inflow as well. This would inhibit further CICR. Additionally to the reaction of the irradiated cell, an intercellular calcium wave in adjacent cells was observed after a delay. It is likely connected to signal transduction via IP_3_ in gap junctions or paracrine signaling via adenosine triphosphate (ATP) [20, 22, 23]. The latter might flow into the medium during perforation as well, which can further induce the intercellular calcium wave. The wave is independent of EGTA, because EGTA does not enter unperforated cells and does not inhibit IP_3_ or ATP signaling. A summary of the different possible processes is given in Fig 7.

### F-actin cytoskeleton after gold nanoparticle mediated laser manipulation

The F-actin cytoskeleton regulates cell shape and generates mechanical force. The actin cortex is connected to the plasma membrane. We aimed to examine the structural and morphological changes in the F-actin distribution of the ZMTH3-Act cells during the first minutes after gold nanoparticle mediated laser manipulation.

**Fig 8:**
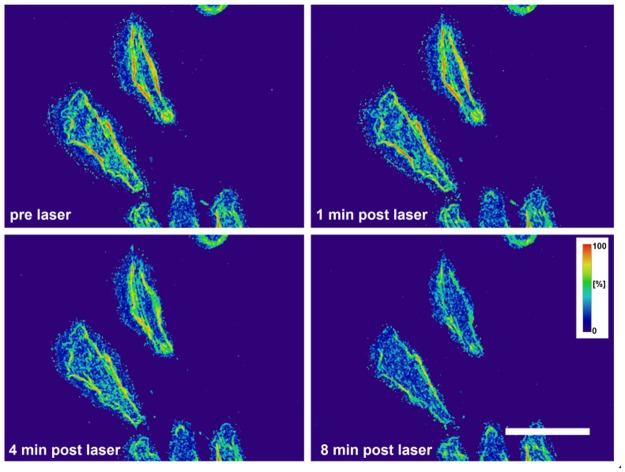
Time series showing the coherency of F-actin in irradiated ZMTH3-Act cells. For visualization a mean filter of three pixels was applied. A coherency of 1 means a high degree of orientation of local image features. A coherency of zero indicates no orientation. Laser parameter: 10 ms irradiation time and radiant exposure of 41 mJ/cm^2^. Scale bar 50 *μ*m.

**Fig 9:**
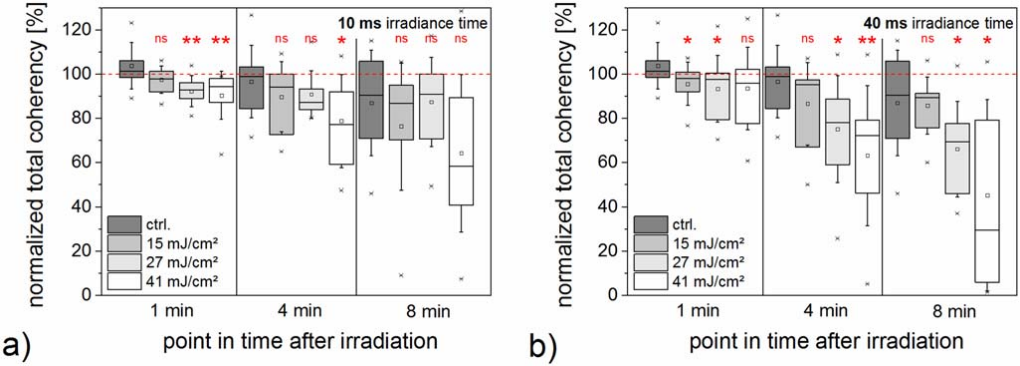
Normalized total coherency of F-actin orientation at distinct points in time after irradiation. Values are normalized to the coherency before laser irradiation. The unirradiated control is based on the same dataset in both diagrams. A disorientation of the F-actin structure is most pronounced for 27 mJ/cm^2^ and 41 mJ/cm^2^ at all three points in time. Additionally, the disorientation increases with time, such that the coherency in the irradiated samples decreases. Data of 10 irradiated cells is shown. Whiskers for box plots depict one standard deviation.

In order to quantitatively address the orientation of the F-actin distribution, we examined the coherency of the F-actin structure in the ZMTH3-Act cells over time (see Fig 8 and Fig 9). Changes in the level of F-actin orientation affect the coherency. For the lowest radiant exposure, the effects on the F-actin structure did not significantly differ from the unirradiated control with one exception at 1 min after perforation at 40 ms irradiation time (see S3 Video). For both irradiation times a slight and unspecific morphological change was visible. In contrast, for higher radiant exposures of 27 mJ/cm^2^ and 41 mJ/cm^2^ a reaction of the F-actin morphology of the cell after exposure was discernible. We examined three different points in time normalized to the F-actin orientation pre laser treatment. One minute after perforation, a radiant exposure of 27 mJ/cm^2^ yielded a slight decrease in the coherency, which tended to even lower values at 4 min and 8 min. This was statistically significant at all points in time at 40 ms irradiation time (*p* < 0.05). After 8 min, the remaining coherency was about 50% with 40 ms irradiation time and a radiant exposure of 41 mJ/cm^2^.

The visualized morphological reactions involved redistribution of F-actin and a measurable loss of orientation of F-actin fibers. This possibly suggests a restructuring process. Chen et al. observed similar tendencies in sonoporation and argue that this could favor membrane repair [37]. This assumption seems valid for cells that are irradiated with low radiant exposures. However, a high loss of orientation might also imply cell death. The measurement time of 10 min also covers cellular phenomena associated with the actin cortex such as blebbing. Blebs expansion lasts about 30 s while retraction takes place in about 2 min [40]. Additionally, some cells showed typical blebbing behavior in this measurement. It might be induced by direct rupture of the cellular actin cortex [46]. This might be related to the observed volume and area change during the first minute and connected to a stress response. However, it might also indicate cell death, as we observed a decrease in cell viability for the higher radiant exposure as indicated in the previous sections.

## Conclusion

In this study, we used a multimodal imaging and manipulation setup to comprehensively measure cell volume and area, calcium concentration changes and F-actin distribution after gold nanoparticle mediated laser manipulation. We applied three radiant exposures at two irradiation times on ZMTH3 cells, which were unspecifically labeled with 200 nm gold nanoparticles. All tested parameters led to successful perforation as evidenced by propidium iodide inflow post perforation. The highest radiant exposure of 41 mJ/cm^2^ and irradiation time of 40 ms primarily led to cell killing. Fast outflow of cellular volume and a pronounced change in cell area were observed in that case. A similar behavior was found by Antkowiak et al. after multiple irradiation doses for femtosecond laser transfection [19]. The cytoplasmatic outflow dominates the inflow in this case. The process was associated with a 50% decrease of total F-actin orientation coherency and a rise in intracellular calcium.

Radiant exposures of 15 mJ/cm^2^ and 27 mJ/cm^2^ resulted in cell viability of more than 70%. The lowest radiant exposure of 15 mJ/cm^2^ did not lead to obvious changes in cell phase volume, area, or F-actin distribution. No change in optical thickness was observed by Antkowiak et al. for standard femtosecond laser transfection parameters as well [19]. A radiant exposure of 27 mJ/cm^2^ led to area reduction. A combination of a fast volume decay process followed by a slow volume change in the first minute after manipulation was observed for the majority of cells. Additionally, cells showed loss of F-actin orientation with a tendency of decreasing orientation for an increasing time after irradiation. Furthermore, highest increase in calcium signal was observed. Calcium response is likely due to inflow of calcium as well as intracellular signaling via the IP_3_ pathway. Additionally, an intercellular calcium wave was observed. These phenomena could be a stress response. This means, that successful gold nanoparticle mediated laser transfection requires a careful choice of parameters. Applying high radiant exposures might possibly produce larger pores which can be crossed by larger molecules, but this study demonstrates that the problem of cellular stress needs to be faced. Furthermore, if a scanning technique is applied to a cell monolayer, stress related signals might be forwarded between all cells, e.g. as an intercellular calcium wave. There is a possibility that a reorganization of the F-actin in combination with a strong volume decrease occurs in dying cells. It is necessary to correlate these parameters to make definitive statements. However, this requires simultaneous measurements of all biophysical and morphological parameters as well as membrane perforation and viability on single cell level for large numbers of cells and irradiation parameters.

In further investigations it is necessary, to extend this knowledge on a more biochemical and molecular basis. It is of great interest to examine heat shock proteins which are key players in cellular stress. Additionally, formation of reactive oxygen species, often associated with oxidative stress, should be investigated. Minai et al. already addressed this issue in the context of femtosecond irradiation of gold nanoparticles [47]. Digital holography has proven a valuable tool for the assessment of volume changes in single cells after gold nanoparticle mediated laser transfection. In further studies it could be fruitful to modify the setup to enable imaging of dense cell monolayers.

In summary, this study demonstrates the necessity of investigating the complex system of cellular response to gold nanoparticle mediated laser manipulation as a whole. Usually the gold nanoparticle mediated laser manipulation and its corresponding perforation process is only studied by investigating its effect on the cell membrane as well as perforation efficiency and viability. By performing a comprehensive analysis after manipulation we could show that the process affects not only the membrane of the cell but also morphological and biophysical parameters such as volume, calcium response and even the F-actin cytoskeleton. By performing further research in this direction, a deeper understanding of underlying biophysical processes can be generated. This could pave the way to open up new applications of gold nanoparticle mediated laser transfection or photothermal therapy.

## Supporting Information

S1 Fig Exemplary fluorescence images for viability and perforation efficiency assessment. In the viability evaluation (a) only green stained cells (calcein) were counted as viable, while red (propidium iodide) or not-green cells (identified using bright field images) were counted as dead. To demonstrate perforation (b), cell nuclei were costained with Hoechst 33342. Propidium iodide is diffused into the cytoplasm and a number of nuclei one minute after perforation. Laser parameters for images shown here are 27 mJ/cm^2^ radiant exposure and 40 ms irradiance time. The laser spot is indicated by an ellipse. Scale bar 30 *μ*m.
(TIF)

S2 Fig Fluorescence brightness of perforated ZMTH3 cells. The fluorescence of a single perforated cell in each measurement from Fig 2 within the laser focus was analyzed directly (a) and one minute after (b) laser irradiation. Three additional radiant exposures are included. Fluorescence imaging was performed using nonlinear gain setting of the camera. The fluorescence was normalized to the highest value. The higher irradiation time enables the inflow of more propidium iodide, additionally the inflow increases with the radiant exposure for each irradiation time. The mean and standard deviation of three independent measurements are shown. Each measurement consisted of thirty irradiated cell areas.
(TIF)

S3 Fig Exemplary graphs of normalized cell phase volume after irradiation for different laser parameters evaluated by holographic microscopy. Cell phase volume is normalized to the average phase volume of the cell before laser manipulation and plotted as a function of time. With higher radiant exposures, the probability of a cell exhibiting an exponential phase volume decay in addition to a linear component increases; compare Fig 3 bottom right box diagram. Data points marked in red correspond to time points before laser irradiation and were not included for fitting. Spikes in the phase volume profile in subfigures c), e) and f) correspond to bubbles in the medium passing over the cell during recording and do not reflect phase volume changes of the cell.
(TIF)

S4 Fig Maximum amplitude and time to peak of the normalized Fluo-4 signal. Only a small variation between EGTA and non-EGTA treated cells is observable. The difference in the time to reach the maximum Fluo-4 signal might be attributed to the absence of calcium inflow in the presence of EGTA in conjunction with EGTA inflow. This is likely to decrease the duration of the Fluo-4 signal. Dataset is based on the same dataset as Fig 6. Whiskers for box plots depict one standard deviation.
(TIF)

S1 Video Exemplary video of cell phase volume change evaluated by holographic microscopy. Images demonstrate the loss of phase volume and area after 40 ms irradiation time for a radiant exposure of 15 mJ/cm^2^ and 41 mJ/cm^2^. A large decrease in area and phase volume is observable in the latter case within 60 s. Scale bar 20 *μ*m.
(MP4)

S2 Video Exemplary video of increase in Fluo-4 fluorescence in the presence and absence of the calcium chelator EGTA. A fixed radiant exposure of 27 mJ/cm^2^ and irradiation time of 40 ms were used. EGTA is a calcium chelator which leads to a less pronounced increase in Fluo-4 fluorescence after laser manipulation. However, an intercellular calcium wave among non perforated cells is still observable. Scale bar 50 *μ*m.
(MP4)

S3 Video Exemplary video of actin changes within 10 min after perforation. A plasmid encodes a marker for the visualization of F-actin in living cells. The cells were irradiated with the given parameters and microscopic images of actin were taken every minute. High radiant exposures led to huge changes in actin and structural rearrangement, while low radiant exposures like 15 mJ/cm^2^ did not cause strong changes. Scale bar 50 *μ*m.
(MP4)

S1 Data Summary data file containing the data of box plots and corresponding statistical analysis. (XLSX)
