# Supplementary figures and images for: Investigation of Biophysical Mechanisms in Gold Nanoparticle Mediated Laser Manipulation of Cells Using a Multimodal Holographic and Fluorescence Imaging Setup
Source: PLoS One. 2015 Apr 24;10(4):e0124052. doi: 10.1371/journal.pone.0124052 (PMC4409398; doi:10.1371/journal.pone.0124052)

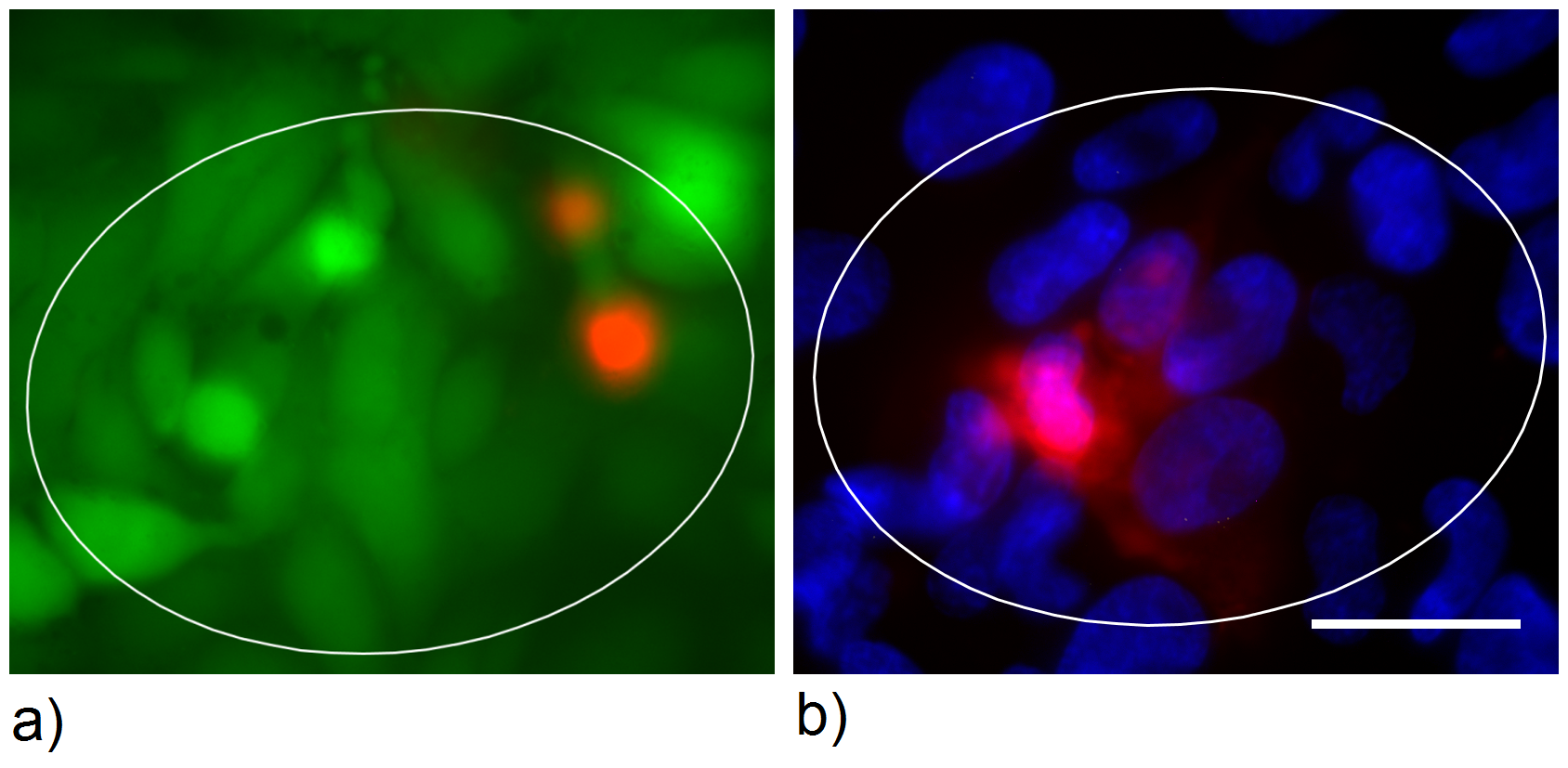

Supplement: S1 Fig — In the viability evaluation (a) only green stained cells (calcein) were counted as viable, while red (propidium iodide) or not-green cells (identified using bright field images) were counted as dead. To demonstrate perforation (b), cell nuclei were costained with Hoechst 33342. Propidium iodide is diffused into the cytoplasm and a number of nuclei one minute after perforation. Laser parameters for images shown here are 27 mJ/cm2 radiant exposure and 40 ms irradiance time. The laser spot is indicated by an ellipse. Scale bar 30 μm. (TIF) [file pone.0124052.s001.tif]

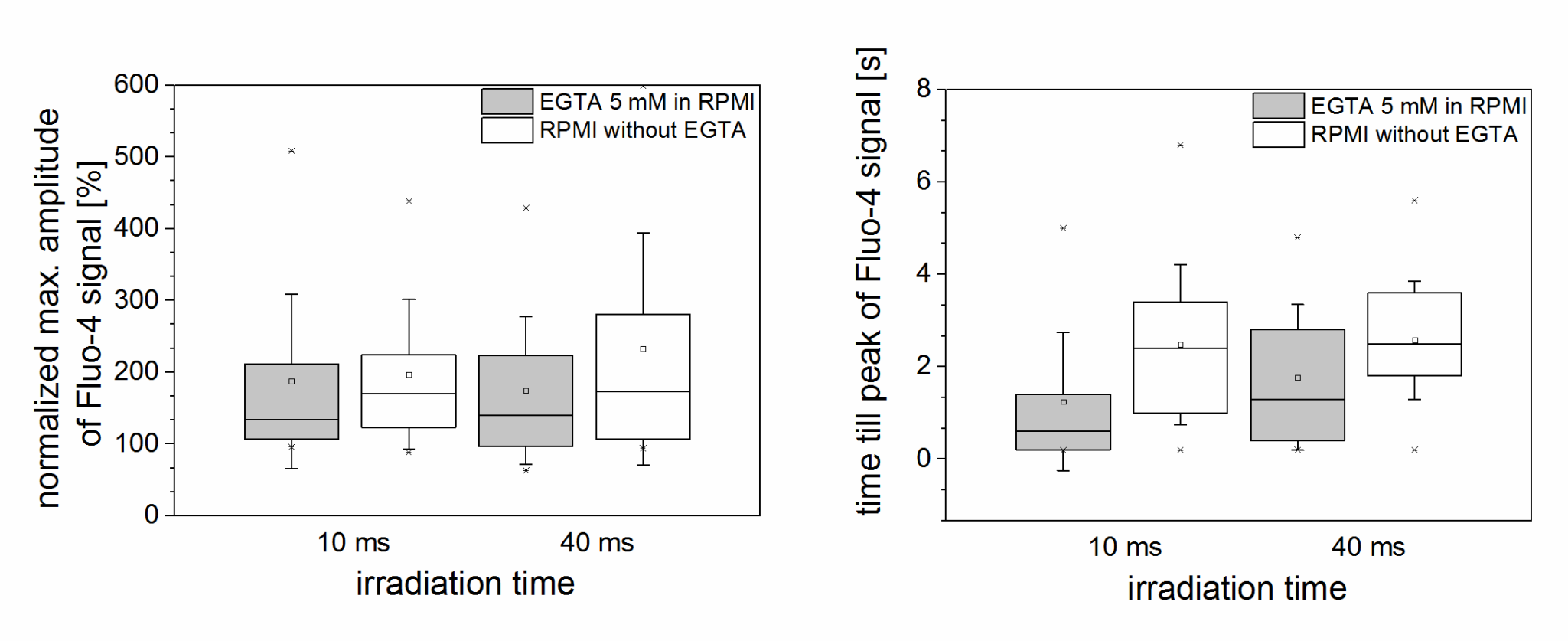

Supplement: S4 Fig — Only a small variation between EGTA and non-EGTA treated cells is observable. The difference in the time to reach the maximum Fluo-4 signal might be attributed to the absence of calcium inflow in the presence of EGTA in conjunction with EGTA inflow. This is likely to decrease the duration of the Fluo-4 signal. Dataset is based on the same dataset as Fig 6. Whiskers for box plots depict one standard deviation. (TIF) [file pone.0124052.s004.tif]
